# Microbially-accelerated consolidation of oil sands tailings. Pathway II: solid phase biogeochemistry

**DOI:** 10.3389/fmicb.2014.00107

**Published:** 2014-03-21

**Authors:** Tariq Siddique, Petr Kuznetsov, Alsu Kuznetsova, Carmen Li, Rozlyn Young, Joselito M. Arocena, Julia M. Foght

**Affiliations:** ^1^Department of Renewable Resources, University of AlbertaEdmonton, AB, Canada; ^2^Department of Biological Sciences, University of AlbertaEdmonton, AB, Canada; ^3^Environmental Science and Engineering, University of Northern British ColumbiaPrince George, BC, Canada

**Keywords:** methanogenesis, Fe^III^ reduction, formation of Fe^II^ minerals, aggregation of clay particles, consolidation, oil sands tailings

## Abstract

Consolidation of clay particles in aqueous tailings suspensions is a major obstacle to effective management of oil sands tailings ponds in northern Alberta, Canada. We have observed that microorganisms indigenous to the tailings ponds accelerate consolidation of mature fine tailings (MFT) during active metabolism by using two biogeochemical pathways. In Pathway I, microbes alter porewater chemistry to indirectly increase consolidation of MFT. Here, we describe Pathway II comprising significant, direct and complementary biogeochemical reactions with MFT mineral surfaces. An anaerobic microbial community comprising Bacteria (predominantly Clostridiales, Synergistaceae, and Desulfobulbaceae) and Archaea (*Methanolinea/Methanoregula* and *Methanosaeta*) transformed Fe^III^ minerals in MFT to amorphous Fe^II^ minerals during methanogenic metabolism of an added organic substrate. Synchrotron analyses suggested that ferrihydrite (5Fe_2_O_3_. 9H_2_O) and goethite (α-FeOOH) were the dominant Fe^III^ minerals in MFT. The formation of amorphous iron sulfide (FeS) and possibly green rust entrapped and masked electronegative clay surfaces in amended MFT. Both Pathways I and II reduced the surface charge potential (repulsive forces) of the clay particles in MFT, which aided aggregation of clays and formation of networks of pores, as visualized using cryo-scanning electron microscopy (SEM). These reactions facilitated the egress of porewater from MFT and increased consolidation of tailings solids. These results have large-scale implications for management and reclamation of oil sands tailings ponds, a burgeoning environmental issue for the public and government regulators.

## Introduction

Aqueous slurries with appreciable clay content (e.g., mine tailings and unconsolidated sediments) are generated worldwide by industrial activities, particularly during ore processing. For example, ~1 million m^3^ of fluid fine tailings day^−1^ are generated during bitumen extraction from surface-mined oil sands ores in northern Alberta, Canada. These wastes are deposited into tailings ponds for containment under a policy of no release to the environment. The implementation of government directives specifying oil sands tailings performance criteria and reduction of tailings volumes has concentrated industry efforts to develop cost-effective technologies for treating these fluid tailings. Strategies include dewatering (recovery of pore water from tailings suspensions for re-use in bitumen extraction) and consolidation of solids (to decrease tailings inventory) after placement in tailings ponds. This poses a major challenge to oil sands companies because gravitational settling of fine colloidal clay suspensions takes years or decades to achieve even 30–35% solids content as mature fine tailings (MFT). Chemical additives (e.g., gypsum) used to consolidate the “fines” can deteriorate the quality of recovered water for re-cycling and can cause unanticipated hazardous side-effects in the ponds (e.g., biogenic H_2_S); physical treatments such as centrifugation are cost- and energy-intensive. Addition of flocculants and coagulants such as polyacrylamide to alter tailings properties (e.g., www.suncor.com/tailings) has unknown long-term stability and environmental impact (e.g., polymer degradation to toxic acrylamide). In contrast, microbially-mediated dewatering and consolidation of fine tailings (“biodensification”) relies on microbes naturally present in tailings ponds (Fedorak et al., 2003), likely supported *in situ* by biodegradation of diluent hydrocarbons (Siddique et al., 2006, 2007, 2011, 2012). In the laboratory, we have accelerated biodensification of MFT by adding fermentable organic carbon sources to stimulate the indigenous microbes: faster dewatering and consolidation were observed with MFT from all five oil sands tailings ponds tested from three different operators, indicating that this is a general response (Bressler et al., 2010).

**Figure 1:**
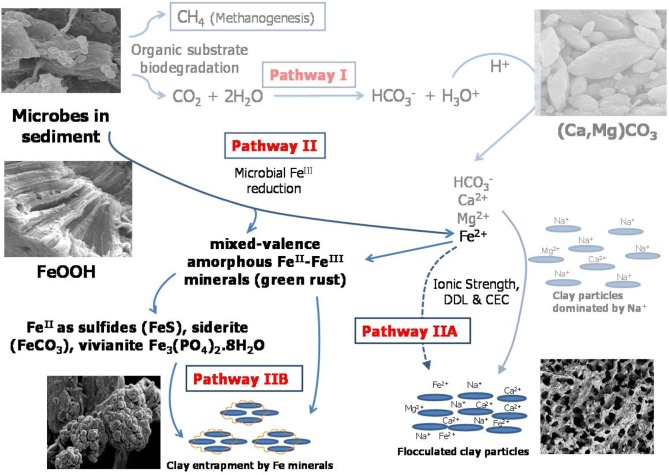
**Proposed model for microbially-mediated geochemical pathways of clay consolidation, modified from Figure 7 in companion paper (Siddique et al., 2014)**. In Pathway I, microbial metabolism decreases pH and dissolves carbonate minerals in MFT, increasing bicarbonate (HCO^−^_3_), calcium (Ca^2+^), and magnesium (Mg^2+^) ions in porewater. These ions increase ionic strength (*I*) of porewater, thus reducing the diffuse double layer (DDL) of clay particles and facilitating their consolidation. In Pathway II under anaerobic conditions, Fe^III^ minerals (goethite and ferrihydrite; FeOOH) in tailings are reduced to Fe^II^ as dissolved Fe^2+^ that may contribute to the cation exchange process (Pathway IIA) and/or to formation of mixed-valence Fe^II^–Fe^III^ “green rust.” Dissolved Fe^2+^ and/or green rust react with H_2_S (aqueous), HS^−^, PO^3−^_4_, or HCO^−^_3_ to form FeS, FeCO_3_, and Fe_3_(PO_4_)_2_¢erdot8H_2_O minerals. Transformed minerals entrap clay particles and/or mask the reactive surfaces of clays, increasing clay particle consolidation (Pathway IIB). Competing reactions are shown by solid arrows while dashed arrows indicate pathways not considered significant in our study. CEC, cation exchange capacity.

To reveal the fundamental mechanisms of biodensification, a 50-L column experiment was conducted to investigate the effects of microbial metabolism on physical, chemical, and mineralogical properties of MFT. We discovered two microbially-mediated geochemical pathways (Figure 1) that increased consolidation and dewatering of MFT. Pathway I, described in a companion paper (Siddique et al., 2014), involves alteration of pore water chemistry via biogenic CO_2_ production, indirectly increasing MFT consolidation. However, those experimental observations also revealed that Pathway I alone was not sufficient to achieve significant tailings consolidation: when exogenous CO_2_ was abiotically purged through the MFT, pore water recovery and MFT consolidation were only slightly increased (Siddique et al., 2014). In contrast, we observed that when biogenic methane and CO_2_ were produced slowly and constantly throughout the tailings during anaerobic metabolism, pore water and clay surface chemistry were altered and physical channels for water transport were created as the organic substrates were depleted. Therefore, in this study we investigated in detail the clay-microbe interactions in MFT samples retrieved after 213 days of incubation, analyzing solid phase chemistry to complement pore water chemistry (Siddique et al., 2014). Based on those results, we propose Pathway II (Figure 1) that is more direct and is complementary to Pathway I to fully describe tailings consolidation and dewatering. This comprehensive study reveals potential microbial repercussions for oil sands tailings ponds management and reclamation strategies, as well as general biogeochemical processes that may influence behavior of unconsolidated clay- and organic-rich sediments such as contaminated riverbeds and harbors.

## Materials and methods

### Preparation and sampling of 50-L columns

An experiment was conducted in 50-L columns using MFT collected from Mildred Lake Settling Basin at Syncrude Canada Ltd. in Fort McMurray, Alberta, Canada (Figure A1). Detailed information about setting up this experiment has been described by Siddique et al. (2014). Briefly, two 50-L acrylic columns were each filled with 45 L MFT under a curtain of N_2_ gas and sealed with an N_2_ headspace to maintain anaerobic conditions. One column contained MFT amended with hydrolyzed canola meal as carbon source (final concentration 400 mg C L^−1^ tailings) to enhance indigenous microbial activity. The canola is not considered a source of viable microbes nor amplifiable DNA because it was hydrolyzed at high pH and 60°C for 24 h. Parallel experiments have not revealed any contributions of microbes from canola amendment to the tailings community (manuscript in preparation). This amendment had been determined previously to promote MFT consolidation at this concentration (Bressler et al., 2010); its preparation is described in Table 2 of a companion paper (Siddique et al., 2014). The other column contained unamended MFT as a baseline control in which only endogenous carbon was available to support microbial activity. The columns were incubated undisturbed in dim light at ~20°C for 213 days. Experimental details and analytical approach for determining biogenic gas production and composition, *in situ* pH, porewater recovery and consolidation of tailings, soluble cations, and anions, exchangeable cations, and carbonates minerals have been described in a companion paper (Siddique et al., 2014). The same samples were subjected to solid phase biogeochemical analyses including fractionation of iron (Fe) and sulfides, mineralogy of solid phase by Scanning Electron Microscopy (SEM) and synchrotron-based X-ray Absorption Near-Edge Structure (XANES) and characterization of microbial communities by 16S rRNA gene pyrosequencing as described below in this section.

### Chemical methods

#### Total metals in MFT solid phase

An acid digestion method (US EPA, 2007) originally designed for sediments, sludge, and soil was used for MFT samples. One gram of solid matrix was separated from MFT by centrifugation under anaerobic conditions, placed in a Teflon digestion vessel and 5 mL H_2_O (nanopure) and 10 mL of 76% HNO_3_ (trace metal grade) were added. Digestion was performed in a microwave (ETHOS SEL High Performance Extraction System) for 20 min at 180°C. After digestion, filtration (0.45 μm pore size) and dilution with 1 % HNO_3_, the samples were analyzed using inductively coupled plasma mass spectrometry (ICP-MS) (PerkinElmer SCIEX ELAN 9000) and results were calculated on a dry weight basis. This acid digestion dissolves almost all metals that could become “environmentally available” (Kimbrough and Wakakuwa, 1989). Elements bound in silicate structures are not normally dissolved by this procedure; however, HNO_3_ treatment with heating can dissolve some clay minerals, e.g., illite and chlorite (Komadel and Madejová, 2006).

For sulfide and Fe fractionation, MFT samples were collected in airtight bottles (Oak Ridge 50 mL centrifuge tubes and Nalgene 250 mL centrifuge bottles with sealing caps) under N_2_ headspace using a continuous stream of N_2_. All further sample manipulations were carried out in “Hands-In-Bag” (Spilfyter, Green Bay WI, USA) under an N_2_ atmosphere. For Fe fractionation, MFT samples were centrifuged (3075 g for 1 h) in a Sorvall RC 5B centrifuge to collect the solids phase whereas whole MFT was used for sulfide fractionation to avoid any H_2_S gas loss during sample manipulation.

#### Sulfide minerals

Different forms of sulfides such as free hydrogen sulfide (H_2_S gas), acid-volatile sulfides (AVS; amorphous and poorly crystalline monosulfides), and chromium (Cr)-reducible sulfides (Cr-R; all sulfides) were determined in the MFT samples. For H_2_S determination, ~3 g of MFT was placed in a pre-weighed vacuum flask under N_2_ atmosphere. The flask was connected by tubing to an Erlenmeyer flask with 30 mL aqueous zinc acetate solution used as a “trap” for H_2_S. The sample was continuously flushed with N_2_ and stirred for 1 h. Free H_2_S was trapped and titrated iodometrically with starch solution as an indicator (Allen et al., 1993; Pansu and Gautheyrou, 2006). Results were reported as sulfur content on a dry weight basis.

Subsequently, for AVS determination, using a syringe 20 mL 6N HCl (Morse and Cornwell, 1987) was injected into the same rubber-stoppered flask and the sample was continuously stirred for 2 h. The digested AVS were trapped in a separate flask containing 30 mL aqueous zinc acetate solution that was analyzed for S by titrating with iodine solution in presence of starch indicator to blue end-point (Ahern et al., 1998; Pansu and Gautheyrou, 2006). Allen et al. (1993) reported that this method was capable of recovering more than 90% of the AVS. AVS usually refers to greigite (Fe_3_S_4_), mackinawite (FeS_0.94_), and amorphous sulfide (FeS) minerals (Ahern et al., 1998; Pansu and Gautheyrou, 2006). In our experimental pH-Eh conditions, mackinawite is assumed to predominate (Rickard and Luther, 2007). Under our experimental conditions the term “amorphous sulfides” includes mackinawite and amorphous sulfide (Rickard and Luther, 2007).

For Cr-R sulfides, about 5 g of MFT was placed in a tared round-bottom three-neck flask under N_2_ atmosphere. The flask was connected through a condenser to an Erlenmeyer flask containing a zinc acetate solution. The sample flask was connected to a N_2_ gas cylinder for continuous flow of N_2_ gas through the apparatus. About 2 g of Cr powder and 60 mL 5.65 M HCl were added to the sample flask. The sample was digested on a hot plate for 1 h (Ahern et al., 1998) then the zinc acetate solution was titrated iodometrically using starch solution as an indicator (Allen et al., 1993; Pansu and Gautheyrou, 2006). Results were calculated as sulfur content on a dry weight basis.

#### Phosphate minerals

For determining Al- and Fe-bound P (mainly Al and Fe phosphates, phosphate adsorbed on Fe oxides and other sorbents, and P in organic components) (Olsen and Sommers, 1982; Kuo, 1996), 1 g MFT was treated with 50 mL 0.1 M NaOH/1 M NaCl solution, shaken for 16 h and centrifuged to separate the supernatant (3075 g for 1 h). The phosphorus in the supernatant was determined using the ascorbic acid method and a UV/VIS spectrophotometer (Optizen POP) at λ 880 nm (Kuo, 1996).

#### Iron (Fe) minerals

Both selective dissolution and direct determination of Fe were used for Fe fractionation in MFT. Total Fe was determined using ICP-MS analysis after acid digestion, as described above for total metals. The ferrozine-extractable Fe^II^ in the solid phase was determined by using a ferrozine method used for the estimation of available Fe^II^ (Sorensen, 1982; Lovley and Phillips, 1986). About 0.3 g MFT solid phase was added to 5 mL of 0.1% ferrozine prepared in a 0.05 M HEPES buffer solution in air-tight tubes under N_2_ headspace. The MFT with ferrozine solution was shaken for 15 min and then centrifuged. The supernatant was analyzed for Fe^II^ using an UV/VIS spectrophotometer (Optizen POP) at 562 nm. To avoid any oxidation of Fe^II^, all MFT manipulations (before addition of ferrozine solution) were performed under N_2_ atmosphere in “Hands-In-Bag” (Spilfyter). To avoid dissolution of accessory minerals by HCl (Wallmann et al., 1993; van der Zee et al., 2002), HCl as an extractant was not used in the analysis.

Dithionite-citrate-bicarbonate (DCB)-extractable Fe was determined by treating the MFT solid phase with DCB solution (pH 7). This method was used to extract the total Fe oxides and hydroxides from soils by reductive dissolution (Mehra and Jackson, 1960; Munch and Ottow, 1980; Pansu and Gautheyrou, 2006). Two grams of MFT solid phase were placed in a 100-mL tube and 45 mL of citrate-bicarbonate buffer (pH 7.3) were added. The sample tube was incubated in a water bath at 75°C and continuously stirred. One gram of sodium dithionite (Na_2_S_2_O_4_) powder was added to the sample when it reached 75°C and a second dose (1 g) was added after 5 min. After 15 min, the sample was centrifuged and the supernatant was transferred to a 250-mL volumetric flask. The sample was treated again with citrate-bicarbonate buffer for a second extraction with two additional doses of Na_2_S_2_O_4_. After centrifugation, both supernatants were pooled and analyzed for Fe by atomic absorption spectroscopy (AAS). DCB extraction dissolves most Fe oxides and oxyhydroxides (both Fe^II^ and Fe^III^) including ferrihydrite (5Fe_2_O_3_. 9H_2_O), lepidocrocite (γ-FeOOH), goethite (α-FeOOH), akaganeite (β-FeOOH), green rust (Fe_3_(OH)_8_), and hematite (αFe_2_O_3_). This extraction also partly dissolves monosulfides (FeS) (Heron et al., 1994) but does not affect siderite (FeCO_3_) and pyrite (FeS_2_) (Lord, 1982; Fine and Singer, 1989; Vodyanitskii et al., 2007). Therefore, DCB extraction is shown to extract both crystalline and amorphous minerals, mostly Fe^II,III^(oxyhydr)oxides.

The ammonium oxalate (AO)-extractable Fe was determined using AO buffer in the dark (AOD), which is commonly used for the determination of amorphous and poorly crystalline Fe oxides and hydroxides in soils (McKeague and Day, 1966; Munch and Ottow, 1980; Pansu and Gautheyrou, 2006) or Fe^III^ in sediments (Phillips and Lovley, 1987). One gram of MFT solid phase was placed in a centrifuge tube with 50 mL of AOD (pH~3). The sample was shaken for 4 h in darkness and centrifuged (3075 g for 1 h), and the supernatant was analyzed for Fe by AAS. Studies have shown that AO extracts crystalline akaganeite and magnetite (FeO·Fe_2_O_3_) (Borggaard, 1988; Heron et al., 1994; Poulton and Canfield, 2005) and some crystalline goethite and hematite, because a significant amount of Fe^II^ extracted in the AO solution catalyzes the dissolution of crystalline oxides (Hering and Stumm, 1990; Phillips et al., 1993). Therefore, AOD extractions may overestimate amorphous Fe^III^ oxides.

Fe^II^ associated with amorphous sulfides (Fe of AVS) was calculated using the molar ratio of Fe to S equivalents (1.7:1) using published approaches (Heron et al., 1994; Kostka and Luther III, 1994).

Fe^II^ associated with pyrite was calculated by establishing the stoichiometric ratio between pyritic sulfide and its equivalent Fe using molar ratios (Fe:S_2_ = 1:1.1). Pyritic sulfide was calculated by subtracting AVS-sulfides from Cr-R sulfides.

Fe^II^ associated with carbonates was calculated after deducting the mass of carbonates associated with Ca and Mg (as CaCO_3_ and MgCO_3_) from the total carbonates. The remaining carbonates were assumed to be associated with Fe, and a molar ratio of Fe:CO_3_ (1:1.07) was used to calculate Fe associated with carbonates.

Fe^II^ associated with phosphates was calculated using the amount of Fe-bound phosphates determined in the preceding section. A molar ratio of Fe:P (2.7:1) was used for the estimation of vivianite (Fe_3_(PO_4_)_2_.8H_2_O).

### Calculating different fractions of Fe in the MFT

#### Fe (total)

Total Fe was determined employing acid (HNO_3_) digestion (US EPA, 2007) using ICP-MS. This method does not dissolve all solid matrices but dissolves almost all compounds that could easily release Fe into the environment, defined as environmentally available Fe. Therefore, the total pool of environmentally available Fe in the MFT samples would be:

(1) $$\text{Fe}(\text{total})={\text{Fe}}_{\text{Acc}.\text{M}}+{\text{Fe}}_{\text{Phyll}}$$

where Fe_Acc.M_ is the Fe of accessory minerals (hematite, magnetite, ferrihydrite, goethite, lepidocrocite, siderite, pyrite, amorphous sulfides, green rust etc., i.e., both Fe^III^ and Fe^II^) while Fe_Phyll_ is the Fe of phyllosilicates. Elements bound in silicate structures are not normally dissolved by this acid digestion method, however HNO_3_ treatment with heating can dissolve some clay minerals, e.g., illite and chlorite. Komadel and Madejová (2006) showed that acid solution (pH<3) even without heating (i.e., at ~20°C) dissolves a portion of phyllosilicates such as smectite and kaolinite.

#### Fe^III^

The following equation was used to calculate Fe^III^ in the MFT:

(2) $${\text{Fe}}^{\text{III}}={\text{Fe}}_{\text{DCB}}-{\text{Fe}}_{\text{ferrozine}}^{\text{II}}$$

Dithionite-citrate-bicarbonate (DCB) extraction (described above) dissolves both crystalline and amorphous minerals such as oxides, oxyhydroxides, and hydroxides (Pansu and Gautheyrou, 2006) but does not dissolve Fe^II^ minerals such as pyrite and siderite. Lord (1982) showed that in pyrite-rich sediment DCB extracted only Fe oxides (at ~75°C), and pyrite was removed only after extraction with 10 M hydrofluoric acid and quartz-distilled nitric acid. Fine and Singer (1989) showed that DCB extraction removed mostly Fe oxide and oxyhydroxide in soil, and fine-grained fractions were removed better than coarse fractions. Vodyanitskii et al. (2007) reported that in soil containing pyrite and siderite, AO-extractable Fe (Fe_AOD_) was greater than DCB-extractable Fe (Fe_DCB_) which showed that AOD dissolved pyrite and siderite while DCB did not dissolve those minerals.

#### Amorphous Fe^III^

The mass of amorphous Fe^III^ was calculated using the following equation:

(3) $$\text{Amorphous }{\text{Fe}}^{\text{III}}={\text{Fe}}_{\text{AOD}}-{\text{Fe}}_{\text{siderite}}^{\text{II}}-{\text{Fe}}_{\text{AVS}}^{\text{II}}-{\text{Fe}}_{\text{ferrozine}}^{\text{II}}$$

Fe_AOD_ usually refers to non-crystalline (amorphous) Fe compounds (Walker, 1983); Fe_siderite_ is Fe associated with carbonates; Fe_AVS_ is associated with gregite (Fe_3_S_4_), mackinawite (FeS_0.94_), and amorphous sulfide (FeS) (Pansu and Gautheyrou, 2006). Under our experimental pH and E_h_ conditions (Eh < −100 mV and pH > 5.5), mackinawite is assumed to predominate (Rickard and Luther, 2007), therefore, we use the term “amorphous sulfide” for both mackinawite and amorphous sulfide minerals (Rickard and Luther, 2007). Fe^II^_ferrozine_ was analyzed using the ferrozine assay (Stookey, 1970) initially developed for Fe^II^ analyses in sediment. Sorensen (1982) and Lovley and Phillips (1986) modified the ferrozine method using HCl to mobilize Fe and hydroxylamine for transforming Fe^III^ to Fe^II^.

AO-buffer at pH ~3.0 also dissolves siderite (Schwertmann and Taylor, 1989; García-Balboa et al., 2011); therefore Fe^II^_siderite_ was subtracted from Fe_AOD_. Amorphous sulfides might also be dissolved for the same reason. Pohl (1962) and Rickard (2006) showed that solubility of FeS increased with decreasing pH. Kostka and Luther III (1994) revealed that AO-buffer could dissolve amorphous sulfides in marine and saltmarsh sediments. Therefore, Fe_AVS_ was subtracted from Fe_AOD_. Ferrozine mobilizes Fe^II^ oxyhydroxides, e.g., green rust, and AOD extraction also dissolves green rust under anoxic conditions (Cornell and Schwertmann, 2007). van der Zee et al. (2002), working with Iberian continental margin sediments, showed that ferrozine did not react with Fe^II^ minerals such as magnetite, monosulfide, siderite, and vivianite. Thus, Fe^II^_ferrozine_ was subtracted from Fe_AOD_. Therefore, amorphous Fe^III^ oxides reported here may be overestimated due to presence of siderite, amorphous sulfides and Fe^II^ oxyhydroxides (e.g., green rust): all these minerals can be dissolved by AO-buffer, therefore the Fe^II^ content of all these minerals was subtracted from Fe_AOD_ to obtain Fe^III^.

#### Crystalline Fe^III^

The crystalline Fe^III^ was calculated using the following equation:

(4) $$\text{Crystalline }{\text{Fe}}^{\text{III}}={\text{Fe}}^{\text{III}}-\text{Amorphous }{\text{Fe}}^{\text{III}}$$

#### Fe^II^

The Fe^II^ was calculated using the following equation:

(5) $${\text{Fe}}^{\text{II}}={\text{Fe}}_{\text{Acc}.\text{M}}^{\text{II}}+{\text{Fe}}_{\text{Phyll}}^{\text{II}}=\text{Fe}(\text{total})-{\text{Fe}}^{\text{III}}$$

#### Crystalline Fe^II^

The crystalline Fe^II^ was calculated using the following equation:

(6) $$\text{Crystalline }{\text{Fe}}^{\text{II}}={\text{Fe}}_{\text{pyrite}}^{\text{II}}+{\text{Fe}}_{\text{vivianite}}^{\text{II}}+{\text{Fe}}_{\text{siderite}}^{\text{II}}$$

Crystalline Fe^II^ comprised the Fe^II^ in dominant Fe^II^-bearing crystalline accessory minerals such as pyrite, vivianite, and siderite that were detected under our experimental conditions. Crystalline Fe^II^ might be underestimated due to the possible existence of some minor crystalline accessory minerals that were not taken into account in our calculations.

#### Amorphous Fe^II^

The amorphous FeII was calculated using the following equation:

(7) $$\text{Amorphous }{\text{Fe}}^{\text{II}}={\text{Fe}}^{\text{II}}-\text{Crystalline }{\text{Fe}}^{\text{II}}$$

### Microscopic and spectroscopic analyses

To observe clay architectural patterns, whole (unmanipulated) MFT samples taken from column ports were cryo-fixed immediately using a cryogenic technique (Emitek K1250 cryogenic system) by plunging them into liquid N_2_. The frozen samples were transferred under vacuum to the cold-stage of the cryo-preparation chamber for ice sublimation (10 min at pressure 10^−5^ Torr). After coating with gold, the samples were transferred into the cryo-SEM chamber, where they remained frozen during SEM imaging.

For conventional SEM and synchrotron XANES spectroscopy, a heavy liquid technique using a hydrophilic sodium polytungstenate solution of Na_6_ (H_2_W_12_O_40_) with density 3 g cm^−3^ (Franke et al., 2007) was used to separate the heavy mineral fraction (density >3 g cm^−3^). Approximately 5 g of MFT solids phase was dispersed in 20 mL Na_6_ (H_2_W_12_O_40_) solution with manual shaking in 50-mL centrifuge tubes. The suspension was centrifuged for 15 min at 3075 g to separate the light and heavy fractions.

For SEM analysis of the heavy fraction to examine mineralogy, samples were coated with carbon using an evaporative carbon coater (Leica EM SCD005). The samples were examined using SEM (JEOL—6301F) with field emission, equipped with a liquid N_2_-cooled lithium drifted silicon X-ray energy-dispersive spectrometer (PGT). XANES was performed to determine the Fe-bearing minerals present in the heavy fraction of the MFT solid phase using Fe K-edge (*E* = 7112 eV). The samples were mounted on Kapton tape (DuPont) and oriented at 45° prior to X-ray beam exposure at the VESPERS beamline in the Canadian Light Source synchrotron facility (Saskatoon, Canada). The scans ranged between 30 eV below and above the absorption edge with a 0.5-eV step size. At least five spectra were collected from each experimental sample and from the reference samples (standards of pure Fe minerals) used in the study. The standard minerals (analytical grade and natural minerals) were purchased from WARD Canada and Sigma-Aldrich (Canada). To avoid oxidation of Fe^II^, strictly anoxic conditions were maintained throughout the entire process of sampling, transport, and preparation of the samples for XANES analysis.

### Microbial characterization by pyrosequencing

The total genomic DNA was extracted from MFT samples using a physical lysis method described previously (Foght et al., 2004). Triplicate 300-μl sub-samples were each extracted twice for a total of six extractions per sample. The precipitated DNA from the six extractions was pooled and used for PCR amplification. A negative control consisting of only extraction reagents was included with every set of samples to ensure contamination was not occurring at any stage of extraction.

After the negative extraction controls had been verified to contain no amplifiable DNA, each genomic DNA extract pool was PCR-amplified in triplicate (3 × 25 μL reactions) using the KAPA2G Robust HotStart DNA polymerase (Kapa Biosystems, Woburn MA). For every triplicate PCR reaction per sample, a negative PCR control was included containing only PCR reagents but no DNA template. Each 25-μL reaction consisted of: 1 μL of genomic DNA, 5 μL of 5X KAPA2G Buffer A, 5 μL of 5X KAPA Enhancer I, 1.25 μL of 100% dimethylsulfoxide, 0.5 μL of a 10 mM dNTP mix, 2 μL of 25 mM MgCl_2_, 2.5 μL of a 2.5 μM working solution of forward primer, 2.5 μL of a 2.5 μM working solution of reverse primer, and 0.5 U of the KAPA2G Robust HotStart DNA polymerase; all reagents were molecular biology grade. The universal primers target both Bacteria and Archaea (An et al., 2013). The forward primer 454T_FB has the 926Fw sequence (AAACTYAAAKGAATTGRCGG) at its 3' end and a B-adaptor sequence (CTATGCGCCTTGCCAGCCCGCTCAG) at its 5' end. The reverse primer 454T_RL_X has the 1392R sequence (ACGGGCGGTGTGTRC) at its 3' end, an L-adaptor sequence (CCATCTCATCCCTGCGTGTCTCCGAC) at its 5' end and a sample-specific 10 nucleotide barcode sequence between adapter and primer, which allows for sample identification after multiplexing during pyrosequencing. The thermocycling program consisted of: one initial 5-min denaturation period at 95°C; 10 cycles of (30 s at 95°C, 30 s at 60°C decreasing by 0.5°C/cycle, and 30 s at 72°C); 30 cycles of (30 s at 95°C, 30 s at 55°C, and 30 s at 72°C); and one final 5 min extension period at 72°C.

Triplicate PCR products from each sample were pooled and an aliquot was examined for quality control by electrophoresis on an agarose gel (i.e., single band of expected size). PCR products were then purified using a QIAquick PCR purification kit (Qiagen) according to the manufacturer's instructions. Purified PCR products were quantified in duplicate using the Nanodrop 2000c (Thermo Scientific) and diluted to 25 ng μL^−1^. Diluted and purified PCR products were again quantified using Nanodrop and visually verified on an agarose gel before being sent to Genome Quebec Innovation Centre, Montreal, QC, Canada for pyrosequencing using a GS FLX Titanium Series XLR70 kit (Roche Diagnostics Corporation). Raw pyrosequencing data were analyzed using Phoenix 2, an in-house developed SSU rRNA data analysis pipeline providing quality control and chimera detection, following the procedure described by Soh et al. (2013). Pyrotag sequences (~7000–10,000 reads per sample) have been submitted to the NCBI Short Read Archive (http://www.ncbi.nlm.nih.gov/sra) under accession numbers SRR621691–SRR621697 representing, respectively: initial, U1, U2, U3, A1, A2, and A3 samples. Quality-verified sequences were compared against the SILVA 102 database (http://www.arb-silva.de) and clustered into Operational Taxonomic Units (OTUs) at 5% distance. OTUs with read abundance <1% in every sample were not considered further in community analysis; hence the total abundance reported is <100%. To compare communities in samples, non-metric multidimensional scaling (NMDS) analysis was implemented in Phoenix 2.

## Results

### Redox conditions, Fe fractionation and mineralogy

Previous small-scale (2-L) experiments demonstrated that stimulating indigenous microbes in MFT with labile organic carbon sources enhanced clay consolidation and pore water recovery (Bressler et al., 2010). In the current experiment we used larger volumes of MFT (45 L) to reduce “wall effects” in the columns, and low concentrations of water-soluble canola meal hydrolysate as an organic carbon source. A parallel unamended MFT control column showed the effects of microbial metabolism of endogenous carbon (e.g., residual hydrocarbons and their metabolites; Siddique et al., 2007). The stationary acrylic columns containing amended or unamended MFT were incubated at ~20°C (circa *in situ* temperature in the tailings ponds) under a N_2_ headspace to maintain anaerobic conditions. As described in a companion paper (Siddique et al., 2014), the MFT underwent bioconsolidation and de-watering measured, respectively, as a decrease in the height of the mudline (the interface between solids and overlying expressed porewater) and an increase in the volume of “cap water” above the mudline; this bioconsolidation was more pronounced in the amended than the unamended MFT column. Three ports in the sides of each column allowed access for measurements and sample collection; Port 1 in each column accessed the cap water whereas Ports 2 and 3 accessed tailings below the mudline; the latter MFT samples comprised porewater (described in the companion paper) and solid phase, described herein.

**Figure 2:**
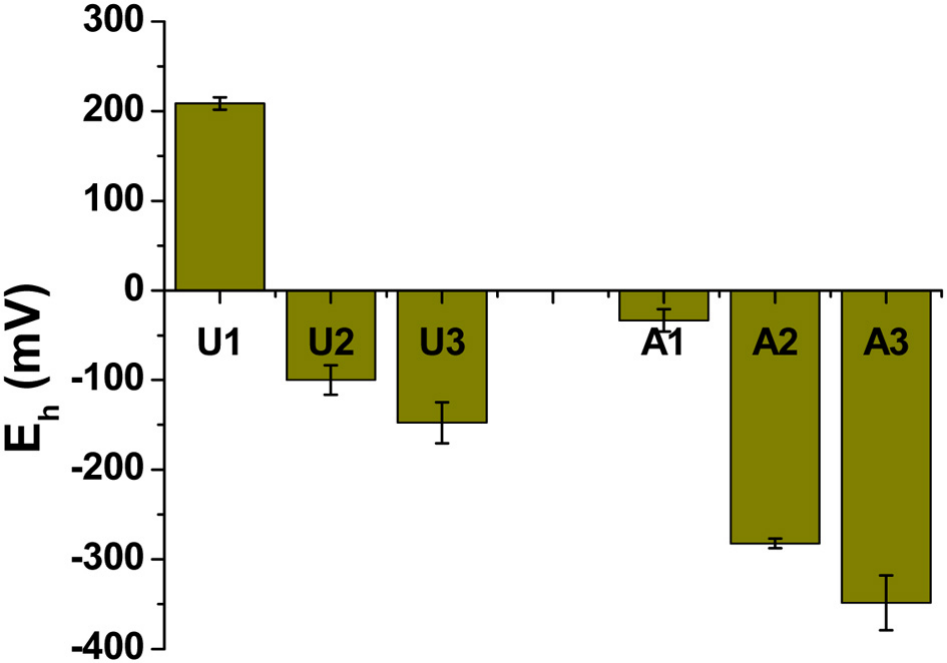
**Redox potential (E_h_) in unamended (U) and amended (A) MFT**. Measurements were taken from port 1 (cap water; see Figure 1 in Siddique et al., 2014) and ports 2 and 3 (solids) after 213 days incubation. Bars represent the mean values from analyses of triplicate samples taken from each column and error bars represent 1 standard deviation.

Methanogenic conditions prevailed in the amended column during incubation, as inferred from the composition of biogenic gas emitted and trapped: by 75 days, amended MFT had generated 2.8 L of CH_4_ and ~0.5 L CO_2_ in the column headspace vs. negligible gas emissions from unamended MFT (Siddique et al., 2014). We did not determine the redox potential (E_h_) of the tailings during incubation because sampling would have disturbed the MFT structure, but we did measure E_h_ when samples were withdrawn at 213 days. Samples from the amended column were more reduced, where E_h_ ranged from −33 mV in cap water to −350 mV at port 3 (Figure 2) vs. the unamended column where suboxic conditions (E_h_ 209 mV) prevailed in the cap water while the MFT below the mudline experienced anoxic conditions with E_h_ of −148 at port 3 (Figure 2).

**Figure 3:**
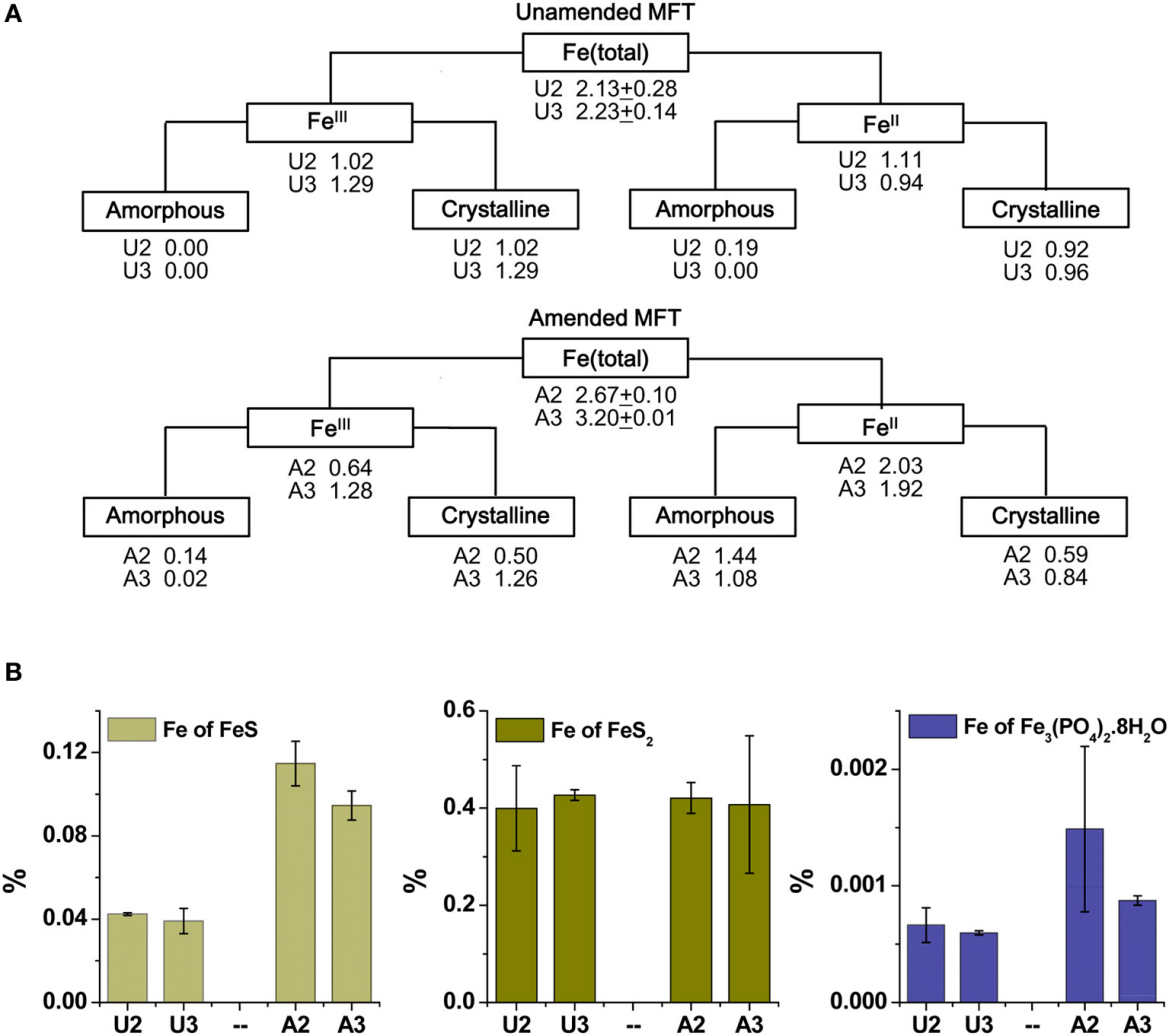
**Iron (Fe) transformation observed in unamended (U) and amended (A) MFT at 213 days incubation**. Labels A2 through U3 designate the columns and ports used to withdraw MFT samples below the mudline. **(A)** Fe fractionation (Table 2) calculated in % (oven dry basis): Fe (total) = Fe (acid digested Fe); Fe^III^ = Fe (dithionite-citrate-bicarbonate; DCB) – Fe^II^ (ferrozine); Fe^III^ (amorphous) = Fe (ammonium oxalate extraction; AOD) − Fe^II^ (siderite) − Fe (acid volatile sulfides; AVS) − Fe (ferrozine); Fe^III^ (crystalline) = Fe^III^ - Fe^III^ (amorphous); Fe^II^ = Fe (total) − Fe^III^; Fe^II^ (crystalline) = Fe (pyrite) + Fe (vivianite) + Fe (siderite); and Fe^II^ (amorphous) = Fe^II^ − Fe^II^ (crystalline). Standard deviations for Fe (total) represent duplicate samples. **(B)** Fe associated with newly formed Fe^II^ minerals (amorphous sulfide [FeS], pyrite [FeS_2_], and vivianite [Fe_3_(PO_4_)_2_.8H_2_O]). Bars represent means of triplicate samples and error bars represent 1 standard deviation.

Because the surface properties of clays are of paramount importance for fines consolidation, minerals in the solids phase of amended and unamended MFT samples collected at Ports 2 and 3 were characterized and quantified. Fractionation and quantitation of Fe in the solid phase is important for inferring its role in microbial metabolism under methanogenic conditions and also for observing transformation (dissolution and precipitation) of minerals under reducing conditions. The forms of Fe in the initial MFT before any manipulation are shown in Table 1. Fe fractionation of the solid phase of unamended and amended MFT samples after 213 days of incubation (Figure 3A) was calculated from the data shown in Table 2 using the approaches described in *Materials and Methods*. In unamended MFT, Fe^III^ and Fe^II^ were present in almost equal concentrations and in crystalline form. Oxyhydroxides were major forms of crystalline Fe^III^ whereas crystalline Fe^II^ comprised pyrite (FeS_2_), siderite (FeCO_3_), and vivianite [Fe_3_(PO_4_)_2_.8H_2_O].

**Table 1:** **Fractionation of iron (Fe) in MFT prior to amendment and incubation**.

**Fe fractions (wt%)^a^; number of replicates**	**Value**
Total Fe^1^ in solid phase; *n* = 2	2.24 ± 0.13
Fe^II^^2^ in solid phase; *n* = 3	0.48 ± 0.02
Fe-DCB^3^ in solid phase; *n* = 2	1.53 ± 0.04
Fe-AOD^4^ in solid phase; *n* = 2	1.11 ± 0.11
AVS^5^ in whole MFT; *n* = 2	0.05 + 0.00
Cr-R sulfides^6^ in whole MFT; *n* = 3	0.34 + 0.05
Iron related P in whole MFT; *n* = 4	0.018 + 0.001

*See Materials and Methods section for detailed description of fractions and analyses*.

a *Calculations were performed on oven dry weight basis*.

1 *Total iron (“environmentally available”) after acid digestion (US EPA, 2007)*.

2 *Ferrozine extractable Fe^II^ in solid phase*.

3 *Dithionite-citrate-bicarbonate extractable iron*.

4 *Ammonium oxalate extractable iron*.

5 *Acid volatile sulfides content*.

6 *Cr-reducible sulfides content*.

**Table 2:** **Iron (Fe) fractionation of unamended and amended MFT after 213 days incubation**.

**Fe fractions (wt%)^a^; number of replicates**	U2^*^	U3^*^	A2^*^	A3^*^
Total Fe^1^ in solid phase; *n* = 2	2.13 ± 0.28	2.23 ± 0.14	2.67 ± 0.10	3.20 ± 0.01
Fe^II^^2^ in solid phase; *n* = 3	0.28 ± 0.09	0.47 ± 0.04	0.30 ± 0.04	0.21 ± 0.02
Fe-DCB^3^ in solid phase; *n* = 2	1.30 ± 0.02	1.76 ± 0.08	0.94 ± 0.05	1.49 ± 0.10
Fe-AOD^4^ in solid phase; *n* = 2	0.80 ± 0.05	0.88 ± 0.03	0.72 ± 0.10	0.75 ± 0.03
Fe of FeS^5^; *n* = 2	0.042 ± 0.001	0.039 ± 0.006	0.115 ± 0.011	0.095 ± 0.007
Fe of pyrite^6^; *n* = 3	0.399 ± 0.087	0.427 ± 0.011	0.421 ± 0.032	0.407 ± 0.142
Fe of carbonates^7^; *n* = 3	0.52	0.53	0.17	0.43
Fe, related to phosphorus^8^; *n* = 3	0.00066 ± 0.00015	0.00060 ± 0.00002	0.00149 ± 0.00071	0.00087 ± 0.00004

*The data presented here were used to construct Figure 3*.

* *U2: port 2, unamended column; U3: port 3, unamended column; A2: port 2, amended column; A3: port 3, amended column*.

1 *Total iron (US EPA, 2007)*.

2 *Ferrozine-extractable Fe^II^ in solid phase*.

3 *Dithionite-citrate-bicarbonate extractable iron*.

4 *Ammonium oxalate-extractable iron*.

5 *Fe^II^ of amorphous sulfides was calculated from AVS content shown in Table 1*.

6 *Fe^II^ of pyrite was calculated from Cr-reducible sulfides and AVS content*.

7 *Fe^II^ of carbonates (FeCO_3_) was calculated after subtracting CaCO_3_ and MgCO_3_ from total carbonates and using stoichiometric ratios*.

8 *Fe^II^ of phosphates (vivianite) was calculated from iron related to phosphorus*.

**Figure 4:**
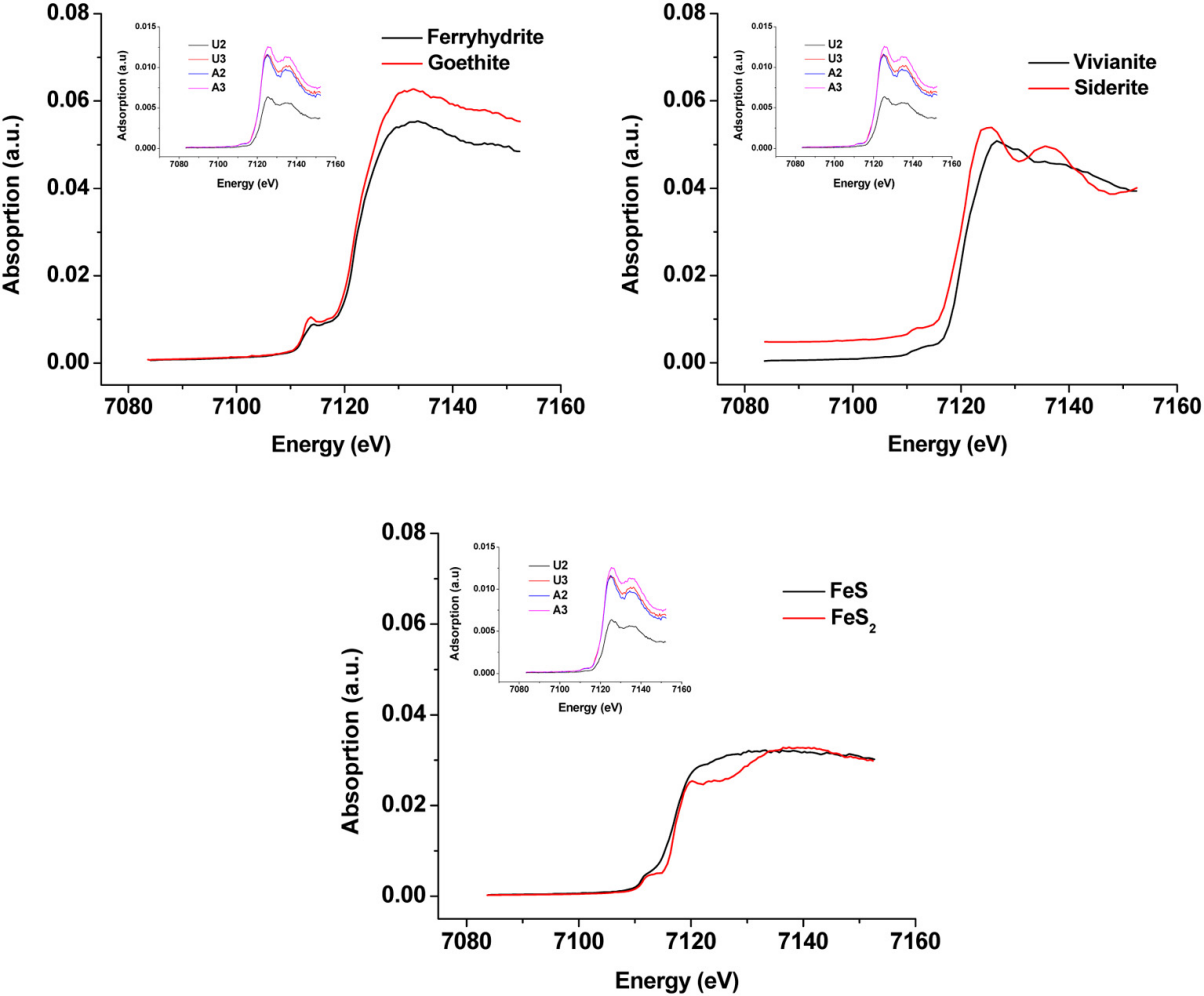
**X-ray Absorption Near Edge Structure (XANES) spectra of unamended and amended MFT from ports 2 and 3 after 213 days incubation (shown as inset in all panels) compared with K-edge XANES of dominant authentic Fe mineral standards (shown as main graphs)**.

Synchrotron analyses (XANES; Figure 4) revealed that ferrihydrite (5Fe_2_O_3_. 9H_2_O) and goethite (α-FeOOH) were dominant Fe^III^ minerals, whereas siderite was the dominant Fe^II^ mineral in both amended and unamended MFT (Table 3). During incubation and microbial metabolism in amended MFT, Fe^III^ decreased and Fe^II^ increased, with amorphous Fe^II^ as the dominant phase. Chemical analyses showed that amorphous FeS and crystalline vivianite increased in amended MFT whereas the concentration of pyrite remained unchanged (Figure 3B). These analyses indicate that significant transformation of iron minerals occurred in the amended MFT during incubation, concomitant with consolidation, de-watering, and gas production (Siddique et al., 2014).

**Table 3:** **Qualitative and semi-quantitative analyses of major iron minerals in unamended and amended MFT after 213 days incubation, determined using K-edge XANES spectra compared with 12 commercial iron mineral standards**.

**Major iron minerals**	U2^*^	U3^*^	A2^*^	A3^*^
Ferrihydrite [Fe(OH)O] ^†^	+++	++	++	+++
Goethite [Fe(OH)O] ^†^	+++	++	++	+++
Siderite [FeCO_3_] ^†^	+++	+++	++++	+++
Vivianite [Fe_3_(PO_4_)_2_] ^†^	++	++	++	++
Pyrite [FeS_2_] ^†^	+	+	+	+
FeS^§^	+	+	+	+
Hematite [Fe_2_O_3_]^†^	−	−	−	−
Magnetite [Fe_3_O_4_]^†^	−	−	−	−
FeO^§^	−	−	−	−
Fe_3_O_4_^§^	−	−	−	−
Fe_2_O_3_^§^	−	−	−	−
FeCl_2_^§^	−	−	−	−

*Athena software (IFEFFIT library for XAFS analysis) was used to perform linear combination fit. Number of + symbols represents estimated relative proportions of minerals. Discrimination among different Fe^III^ oxyhydroxides is difficult using XANES (Prietzel et al., 2007)*.

* *U2: port 2, unamended column, U3: port 3, unamended column, A2: port 2, amended column, A3: port 3, amended column*.

† *Research minerals (Ward's Science Canada)*.

§ *Reagent grade chemicals (Sigma-Aldrich, Fisher Scientific)*.

### Clay architecture

**Figure 5:**
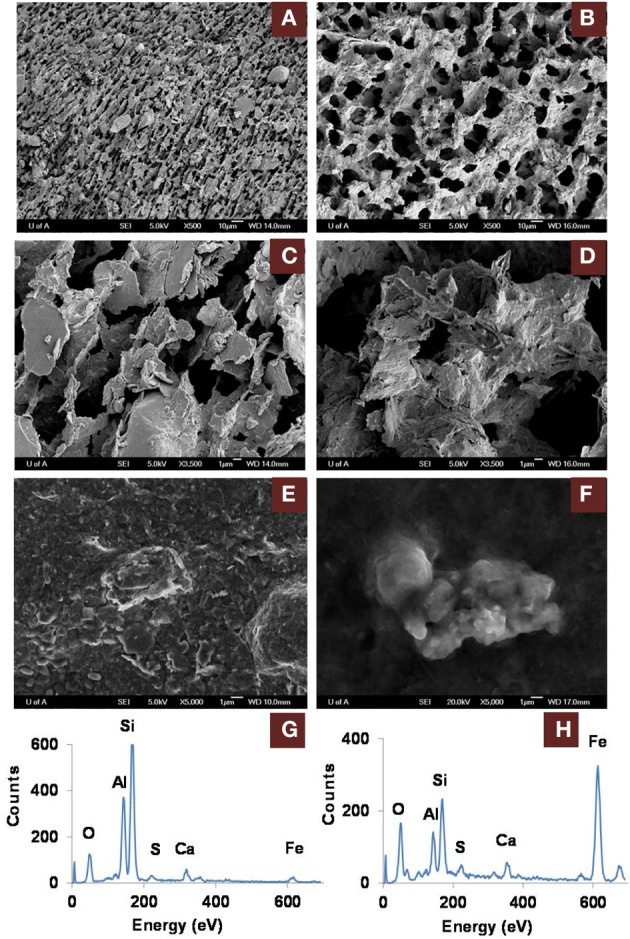
**Scanning electron microscope (SEM) micrographs of clay architecture in amended and unamended MFT at 213 days incubation. (A–D)**, cryo-SEM; **(E,F)**, conventional SEM. **(A)** Unamended MFT with random clay particle structure; scale bar 10 μm. **(B)** Canola-amended MFT with flocculated clay particles (light) having card-house structure, producing a network of interstitial pores (dark); scale bar 10 μm. **(C)** Phyllosilicate particles in unamended MFT, lacking transformed mineral coating; scale bar 1 μm. **(D)** Phyllosilicate particles in amended MFT, forming aggregates with amorphous surface coating; scale bar 1 μm. **(E)** Weakly aggregated phyllosilicate particles in unamended MFT. **(F)** Aggregated phyllosilicate particles in amended MFT. **(G)** Energy dispersive spectrum (EDS) of **(E)** showing phyllosilicates with negligible iron mineral coating. **(H)** EDS of **(F)** revealing phyllosilicates coated with amorphous iron minerals.

The greater recovery of porewater from amended vs. unamended MFT (Siddique et al., 2014) led us to use cryo-SEM to examine the solid phase and determine the clay architecture of MFT samples collected at 213 days of incubation (Figure 5). Microscopy revealed aggregation of clay particles in the amended MFT, producing networks of pores between aggregates that presumably would facilitate egress of pore water and biogenic gas from MFT (Figure 5B), compared with the fine pore structure preserved in the unamended MFT (Figure 5A). Newly transformed amorphous Fe minerals coated the charged clay surfaces in the amended MFT and the coatings observed on aggregate surfaces in amended MFT (Figure 5D) were even more apparent using conventional SEM (Figure 5F). Energy dispersive spectroscopy (EDS) analyses performed during cryo-SEM confirmed that the clay aggregates in amended MFT were coated with amorphous Fe minerals (Figure 5H) that, according to mineralogical analyses described above (Figure 3, Table 3), were newly formed by transformation of crystalline iron minerals during incubation. In the unamended MFT, clay particles were observed randomly oriented and closely stacked, without any Fe coating (Figures 5A,C,E,G). These observations imply that transformation of iron minerals has changed the clay surfaces, allowing them to create larger pore structures stabilized by formation of new minerals.

### Microbial community structure

**Figure 6:**
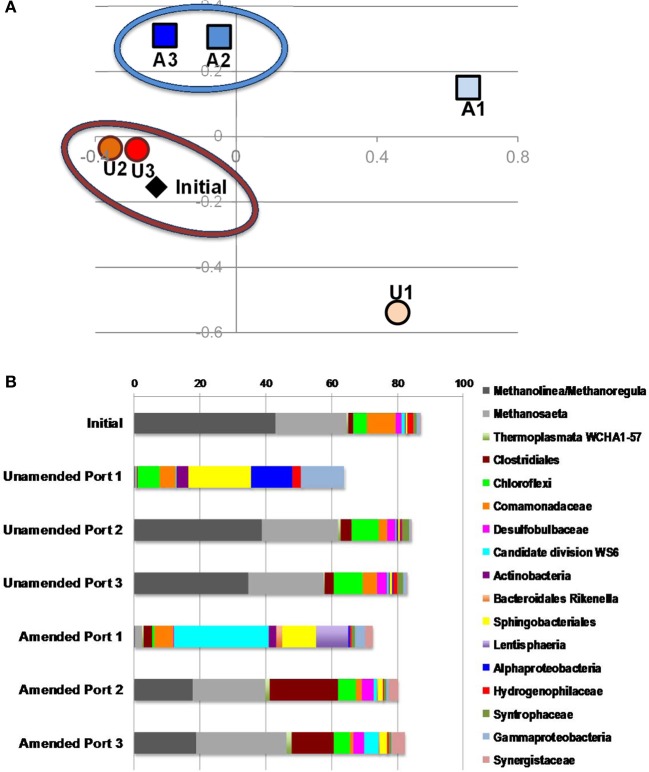
**Microbial community analysis. (A)** Non-metric multidimensional scaling (NMDS) analysis of 16S rRNA gene pyrosequences comprising microbial communities in initial MFT (black diamond) and MFT samples taken from unamended (U; orange circles) and amended (A; blue squares) column ports. Numbers indicate the ports (Figure 1 in Siddique et al., 2014), with port 1 accessing cap water and ports 2 and 3 accessing MFT in both columns. **(B)** Microbial community composition in the initial bulk MFT, cap water (port 1) and tailings (ports 2 and 3) in the unamended and amended MFT after 213 days incubation, based on 16S rRNA gene pyrosequencing. Only OTUs present at an abundance of ≥1% in one or more communities were considered; hence the total community is <100%.

To understand electron flow from labile organic carbon to Fe^III^ minerals in MFT via indigenous microorganisms, 16S rRNA gene pyrosequencing was performed. NMDS analysis was performed on the total communities in initial MFT (before manipulation) and in unamended and amended MFT samples taken from Port 1 (the water cap) and Ports 2 and 3 (tailings below the mud line) after 213 days incubation. Figure 6A shows that the unamended tailings communities were most similar to the initial MFT, whereas the amended tailings communities and both cap water communities differed from the original tailings communities and from each other.

The microbial community initially present in MFT before manipulation comprised a majority of methanogens (~65% of pyrosequences) dominated by putative hydrogenotrophs (43% *Methanolinea/Methanoregula*), with fewer acetoclastic methanogen sequences (~20% *Methanosaeta)* (Figure 6B). The bacterial component included Betaproteobacteria (family Comamonadaceae; 9%) and Chloroflexi (4%) as dominant phyla, with minor contributions by diverse bacterial taxa. After 213 days incubation, the community in unamended tailings below the mudline (Ports 2 and 3) still closely resembled the initial MFT community. However, Port 1 in the unamended column accessed the expressed pore water that collected above the mudline. The community structure of that cap water had shifted radically, being virtually depleted of methanogens but enriched in anaerobic Bacteroidetes (order Sphingobacteriales) and in Alpha- and Gammaproteobacteria, which include numerous facultative anaerobes. The proportion of reads assigned to “rare” taxa (each with abundance <1%) comprised nearly 40% of total reads, with virtually all being bacterial.

The microbial community detected in amended MFT solids also shifted during incubation, compared to the initial and the unamended solids. Fewer hydrogenotrophic methanogen sequences (*Methanolinea* and *Methanoregula*) resulted in a lower proportion of total methanogenic sequences, but the proportion of acetoclasts (*Methanosaeta*) was almost unchanged (Figure 6B). Among bacterial sequences, the diversity and abundance of Clostridiales (particularly *Anaerobacter*) and Synergistaceae (including *Thermanaerovibrio)* increased significantly in amended vs. initial and unamended MFT (Table 4), likely reflecting increased fermentation of the hydrolyzed canola meal amendment. In contrast, betaproteobacterial sequences (mainly in the family Comamonadaceae) decreased in amended MFT solids compared to initial MFT. The community in cap water overlying amended MFT diverged substantially from the MFT solids with a precipitous decline of archaeal sequences that were replaced by sequences affiliated with the uncharacterized, uncultivated candidate division W6. Sequences of the order Sphingobacteriales and little-known class Lentisphaeria also increased in abundance. The “rare” microbiome (each <1% abundance) comprised about one-third of the total pyrosequences. Thus, amendment shifted the MFT solids community slightly toward fermenters while retaining a substantial proportion of methanogens (>40%), but expression of pore water radically altered the community structure to become more diverse, no longer resembling the solids-associated community.

**Table 4:**
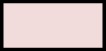
**Relative abundance of 16S rRNA gene pyrosequences affiliated with the bacterial orders Clostridiales and Synergistales in MFT samples prior to amendment and in amended and unamended MFT after 213 days incubation**.

^*^ Color highlight indicates abundance of total reads: –, not detected, 
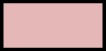
*0.1-0.5%*
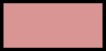
*0.5–1%*
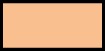
*1–2%*
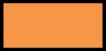
*2–3%*
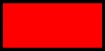
*3–4%*
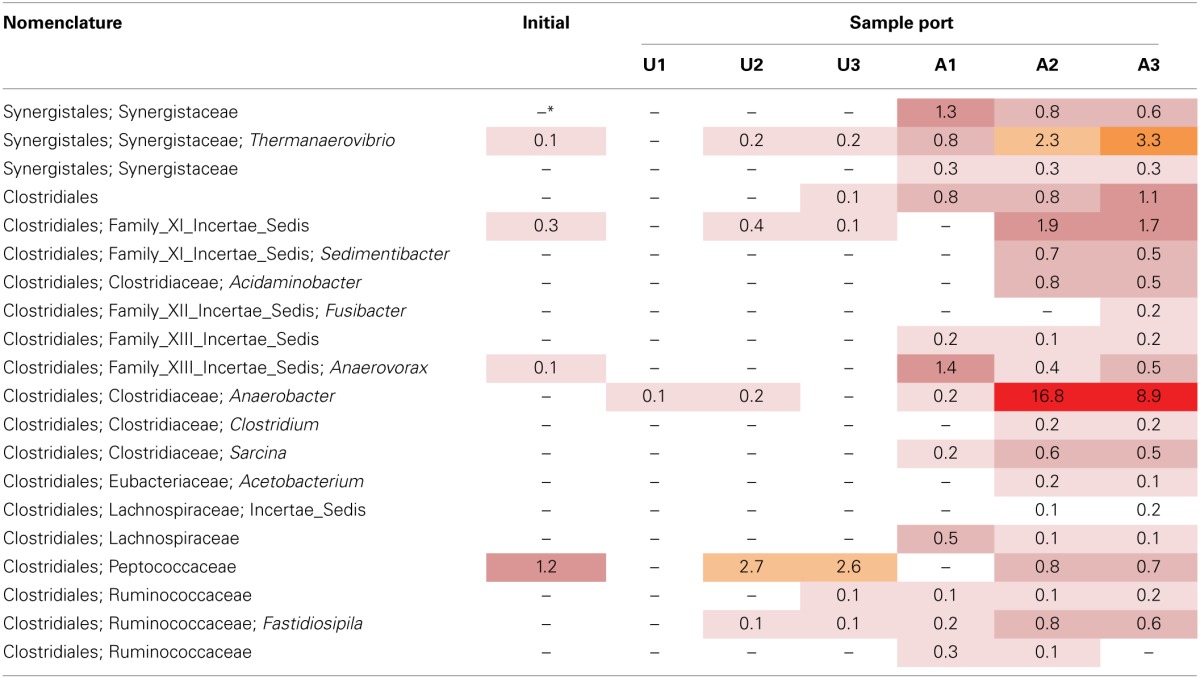
>*4*.

## Discussion

In a companion paper we described how microbial activity in oil sands tailings, including methanogenesis, promoted rapid consolidation of clay particles, (Siddique et al., 2014). Pathway I (Figure 1) proposes that the indigenous microbial tailings community alters pore water chemistry by producing CO_2_, which dissolves in pore water and lowers its pH. The decrease in pore water pH increases dissolution of carbonate minerals, which increases the ionic strength of the porewater. This in turn reduces the diffuse double layer (DDL) of clay particles and enhances consolidation of oil sands tailings. The other important and direct biogeochemical mechanism influencing consolidation of oil sands tailings is microbial reduction of Fe^III^ minerals (Pathway II; Figure 1), discussed in the current study. Pathway II invokes microbial reduction of Fe minerals in amended MFT during organic carbon metabolism under methanogenic conditions.

Fe undergoes redox transformation processes linked to microbial metabolism (Roden, 2012). Comparing Fe fractionation in amended and unamended MFT after 213 days of incubation, we observed that microbial metabolism transformed Fe^III^ minerals so that more amorphous Fe^II^ minerals appeared in amended MFT. Chemical analyses showed that amorphous FeS and crystalline vivianite increased in amended MFT. Because amorphous FeS constitutes only 10–14% of total amorphous Fe^II^ in amended MFT, we propose that goethite and ferrihydrite transformed into green rust (mixed-valence Fe^II^-Fe^III^ hydroxides, carbonates and/or sulfates), part of which was further transformed into other Fe^II^ minerals such as sulfides, siderite, and vivianite. Green rust is a mixed-valence amorphous Fe^II^-Fe^III^ mineral. Jiang et al. (2013) revealed that Fe^III^ mineral was transformed to soluble Fe^II^ that was re-adsorbed and re-precipitated as Fe^III^ after donating electrons to methanogens during CH_4_ production. Owing to the great variation in the composition of green rust (Cornell and Schwertmann, 2007) and unavailability of a suitable chemical method to analyze it, we did not quantify green rust in MFT but speculate on its occurrence based on mass balance calculations and observations in other systems where goethite and ferrihydrite transformation occurred, forming the green rust minerals fougerite [Fe^II^_2_Fe^III^(OH)_7_] in fresh water and GR2 [Fe^II^_4_Fe^III^_2_(OH)_12_SO_4_] in sea water at pH 7–9 (Rickard and Luther, 2007). However, we cannot exclude contributions from abiotic Fe^III^ reduction because Fe^II^ also catalyzes reduction and dissolution of Fe^III^ minerals (Suter et al., 1988; Kostka and Luther III, 1994), including ferrihydrite and goethite transformation into mixed Fe^II^–Fe^III^ green rust at pH 6.3 (Usman et al., 2012).

Formation of Fe^II^ minerals such as sulfides, siderite, and vivianite in amended MFT occurred either through transformation of green rust group minerals, which are considered the most significant precursors (Fe source) of sulfide minerals (Rickard and Luther, 2007), or directly through dissolved species such as Fe^II^ (Fe^2+^), sulfide (S^2−^, HS^−^), CO^2−^_3_ and PO^3−^_4_ where Fe^2+^ and S^2−^ were produced during microbial reduction of Fe^III^ minerals and SO^2−^_4_, respectively. The MFT contained low concentrations of SO^2−^_4_ (for FeS precipitation) and PO^3−^_4_ (for vivianite formation) but more HCO^−^_3_ was available for siderite formation [Table 1 and Figure 6; (Siddique et al., 2014)]. We did not detect Fe^2+^ in porewater or on clay surfaces in any columns using the described methods. Therefore, we postulate that Pathway IIA (Figure 1) does not contribute significantly to the consolidation of tailings but that Fe^2+^ was converted to Fe^II^ minerals that entrapped clay particles (Pathway IIB).

**Figure 7:**
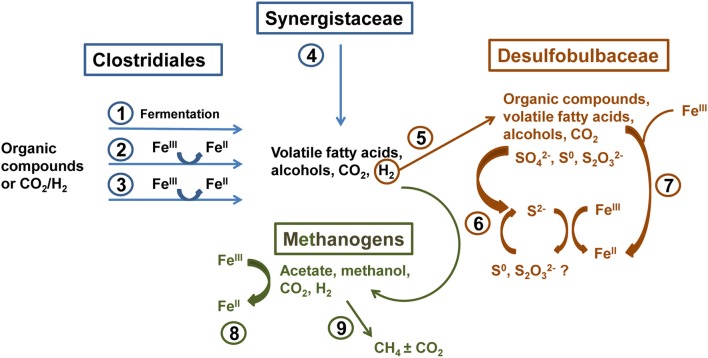
**Proposed biogeochemical reduction of Fe^III^ and cycling of S in amended MFT, based on enrichment of Clostridiales, Synergistaceae, and Desulfobulbaceae in amended MFT as revealed by 16S rRNA gene pyrosequencing (Figure 6). (1)** Clostridiales ferment organic substrates yielding fatty acids, alcohols, CO_2_, and H_2_. **(2)** Fermentative metabolism can divert a proportion of electron flow to Fe^III^ reduction without conserving energy for growth (Coleman et al., 1993; Dobbin et al., 1999). **(3)** Some Clostridia reduce Fe^III^ through respiration (dissimilatory reduction) to support growth (Slobodkin et al., 1999; Kunapuli et al., 2007). **(4)** Synergistaceae ferment organic substrates (Zavarzina et al., 2000). **(5)** H_2_ produced by Clostridia and Synergistaceae during fermentation can be utilized syntrophically by sulfate-reducing bacteria (SRB; e.g., Desulfobulbaceae) (Kunapuli et al., 2007). **(6)** some SRB can indirectly reduce Fe^III^ via S cycling (Straub and Schink, 2004), biogenic S^2−^ can chemically reduce Fe^III^ minerals (Raiswell and Canfield, 1998; Poulton et al., 2004), and/or **(7)** SRB can directly transfer electrons to Fe^III^ (Knoblauch et al., 1999). **(8)** Methanogens oxidizing H_2_ can reduce Fe^III^ by transferring electrons to Fe^III^ both directly and via electron shuttling (Bond and Lovley, 2002; Liu et al., 2012). **(9)** Hydrogenotrophic and acetoclastic methanogens produce CH_4_ syntrophically with Bacteria in MFT (Siddique et al., 2011, 2012).

Molecular analysis of microbial communities in MFT samples suggests that Fe transformation in amended MFT was not carried out by canonical Fe-reducing bacteria. Microbial communities in amended MFT comprised ~50% methanogens with a slightly higher proportion of acetoclastic methanogens (*Methanosaeta*). Among the bacterial sequences that constituted ~50% of microbial communities, the abundance of members of the order Clostridiales and family Synergistaceae increased significantly in amended vs. initial and unamended MFT. Microbial characterization led us to propose a microbially-mediated Fe^III^-reduction pathway (Figure 7) in which Clostridiales and Synergistaceae ferment organic carbon in amended MFT to produce simple fatty acids, alcohols, CO_2_, and H_2_ as substrates for sulfate-reducing bacteria (SRB) such as Desulfobulbaceae, and for methanogens via syntrophic interactions. Fermenters do not completely oxidize fermentable substrates to CO_2_ when Fe^III^ is the sole electron acceptor but they can divert a portion of the electrons from fermentation to Fe^III^ as an electron sink or supplementary electron acceptor without conserving sufficient energy for cell growth (Coleman et al., 1993; Dobbin et al., 1999). Other studies support these observations, with Clostridiales and Desulfobulbaceae being involved in Fe^III^-reduction during benzene degradation (Kunapuli et al., 2007). Jiang et al. (2013) reported that syntrophic acetate-oxidizing Clostridia oxidized acetate to HCO^−^_3_ and H^+^, reducing Fe^III^ mineral (akaganeite) to soluble Fe^II^, which donated electrons to methanogens to produce CH_4_ by utilizing HCO^−^_3_ and H^+^. Whether Desulfobulbaceae in MFT directly reduce Fe^III^ or shuttle electrons to Fe^III^ through S^2−^/S^0^ cycling (Straub and Schink, 2004) could not be discerned here. However, the role of biogenic S^2−^ in chemical reduction of Fe^III^ minerals (Raiswell and Canfield, 1998; Poulton et al., 2004) is not considered significant in our study because of the low initial concentration of SO^2−^_4_ in MFT (Penner and Foght, 2010) and the formation of only small amounts of amorphous FeS during incubation.

Methanogens that reduce CO_2_ using H_2_ can also transfer electrons to Fe^III^ either directly or through electron shuttles like extracellular quinones (Bond and Lovley, 2002; Liu et al., 2011). Solid-phase humic substances could also shuttle electrons from bacteria to Fe^III^ oxide minerals (Roden et al., 2010). Thus, humic acids and other soluble organic fractions in MFT (Majid and Ripmeester, 1990) may serve as electron shuttles for Fe^III^ reduction in MFT. This inadvertent extracellular Fe^III^ reduction squanders potential energy, possibly explaining the diminished abundance of hydrogenotrophic methanogens in amended MFT (Figure 6B). Considering the proportions of microbial groups detected in MFT, we postulate that Clostridia, Synergistia, Desulfobulbaceae, and methanogens are the key players in Fe^III^ reduction in amended MFT (Figure 7, pathways 1–4 and 8).

Clay aggregate formation (Pathway IIB, Figure 1) is a primary process for dewatering and consolidation of tailings (Brown et al., 2013). The aggregation of clay particles and formation of networks of pores explains the recovery of more water from amended MFT. Clay particle aggregation is facilitated by the formation of amorphous Fe minerals that trap and coat the charged clay surfaces. The combined results of this study (Figure 1; Pathways I and II depicting changes in pore water and solid phase chemistry, respectively) reveal that microbially-mediated geochemical processes affect aggregate structure and hence the rate of consolidation. Decreasing pH favors formation of face–edge structures in kaolinite (the dominant clay in MFT) by protonating aluminum and silanol groups (Si-O) exposed at the edges of kaolinite plates, thus attracting the negatively charged faces of the plates to form “house-of-cards” structures. At higher pH, both the edges and faces of clay particles are negatively charged and dispersed. Compression of DDL by increasing *I* (at both high and low pH) causes particles to adhere along basal surfaces (face–face) to form “deck-of-cards” structures that generate thicker aggregates having higher density (Nasser and James, 2006; Zbik et al., 2008; Mietta et al., 2009) and decreased mobility of interstitial water. Because previous studies (Nasser and James, 2006; Zbik et al., 2008; Mietta et al., 2009) were performed using pure phyllosilicates rather than the complex mixture of phyllosilicates, iron oxyhydroxides, and organics comprising MFT, we cannot unequivocally extrapolate to the flocculation structures prevalent in amended MFT (Figures 5A–D). However, compared to the dispersed clay particles in unamended MFT (Figures 5A,C), well-structured flocculation was observed in amended MFT (Figures 5B,D), apparently dominated by card-house stacking similar to the coagulated card-house structure observed in sulfidic sediments (Macdonald et al., 2009) rather than the card-deck structure that limits dewatering of tailings (Zbik et al., 2008). Based on the current study, we proposed a model (Figure 1) describing the mechanisms encompassing various biogeochemical processes occurring simultaneously in MFT that culminate in microbially-mediated consolidation of oil sands tailings.

Our proposed mechanisms of MFT consolidation are supported *in situ* by measurement of more rapid MFT consolidation (>15-fold faster rates) in areas of Mildred Lake Settling Basin (the largest and one of the oldest oil sands tailings ponds, and the source of MFT used in this study) having well-established methane production (Fedorak et al., 2003). This pond continuously receives tailings containing labile hydrocarbons that sustain active methanogenic communities (Penner and Foght, 2010; Siddique et al., 2011, 2012). We have also consistently observed the phenomenon of microbial consolidation of fines using oil sands tailings collected from three other oil sands operators (unpublished results), even though the tailings differ in their mineralogy, nature of extraction solvents and age of deposition.

This study uncovers complex, interwoven biogeochemical pathways that impact environmental and reclamation issues associated with oil sands tailings. Microbially-enhanced tailings consolidation can decrease on-site freshwater demand for processing oil sands ores by accelerating porewater recovery from the ponds for re-use. Biodensification may also facilitate dry landscape reclamation of MFT by complementing or possibly foregoing current management practices like chemical flocculant addition and energy-intensive centrifugation processes. However, transformation of clay minerals *in situ* can influence distribution of soluble metals and affect the quality of cap water associated with tailings ponds. The synthesis of new minerals such as iron sulfides may also affect the acid production potential of tailings used in surface land reclamation scenarios. Thus, the implications of this study are important for constructing and managing oil sands tailings ponds and designing future reclamation options. More broadly, microbial contributions to consolidation of diverse organic-rich clay suspensions should be investigated and incorporated into geotechnical models.

### Conflict of interest statement

The authors declare that the research was conducted in the absence of any commercial or financial relationships that could be construed as a potential conflict of interest.
