# Supplementary material for: Microbially-accelerated consolidation of oil sands tailings. Pathway II: solid phase biogeochemistry
Source: Front Microbiol. 2014 Mar 21;5:107. doi: 10.3389/fmicb.2014.00107 (PMC3968759; doi:10.3389/fmicb.2014.00107)
Supplement: Supplementary file 1 [file Presentation1.PDF]

APPENDIX

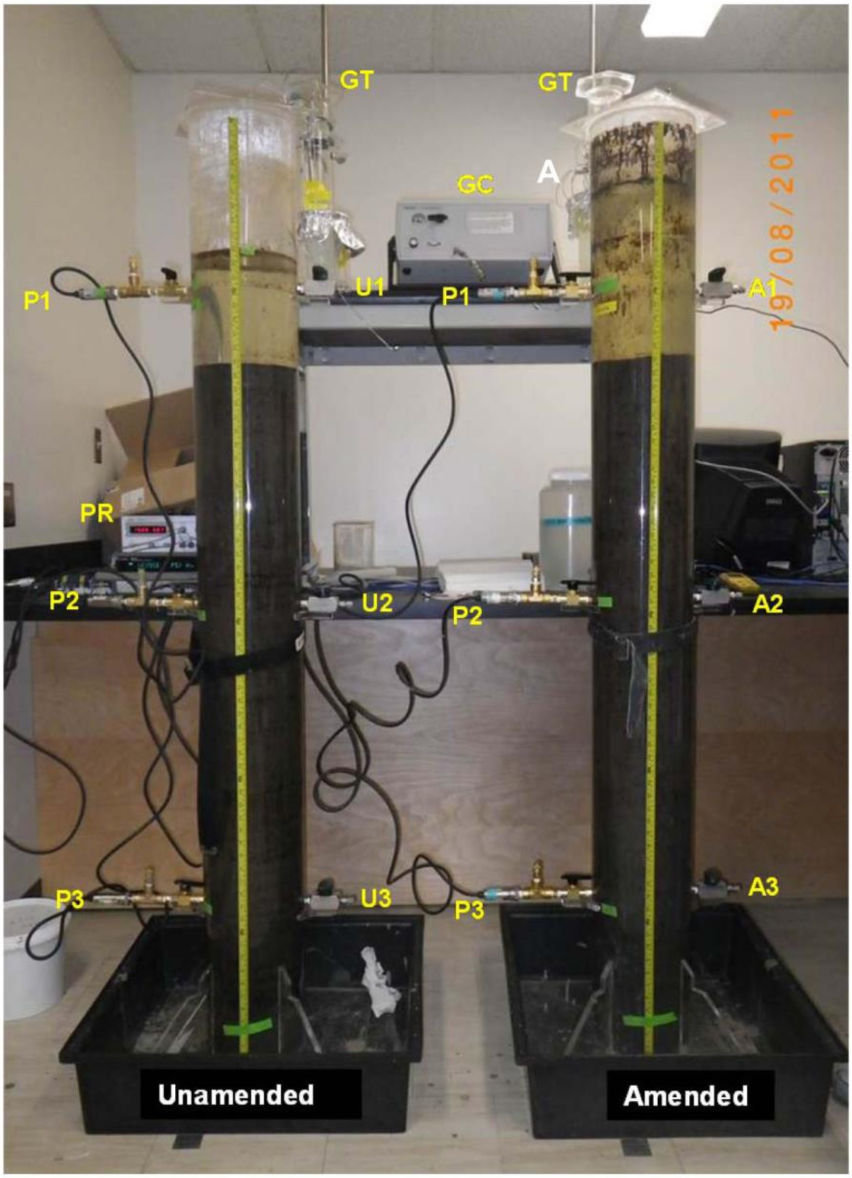

Fig. A1: Experimental 50-L columns used for measuring consolidation, porewater recovery and gas release from oil sands mature fine tailings (MFT) either amended (A) with an organic carbon source or unamended (U). The photograph was taken after 105 d incubation at ~20°C. Three sampling ports for collecting MFT or cap water (A1–A3; U1–U3) and three instrumentation ports (P1–P3) fitted with pressure transducers and a pressure reading unit (PR) for recording in situ pore pressure were installed in each column; see Methods for details. The columns were sealed under a N_2 gas atmosphere. Stainless steel tubing connected the headspace to a micro-gas chromatograph (GC) to determine the composition of released biogenic gas, and to gas traps (GT) for measuring cumulative gas production volumes.
